# Aerosol formation during processing of potentially infectious samples on Roche immunochemistry analyzers (cobas e analyzers) and in an end-to-end laboratory workflow to model SARS-CoV-2 infection risk for laboratory operators

**DOI:** 10.3389/fpubh.2022.1034289

**Published:** 2022-11-16

**Authors:** Géza V. Burghardt, Markus Eckl, Doris Huether, Oliver H. D. Larbolette, Alessia Lo Faso, Beatus R. Ofenloch-Haehnle, Marlene A. Riesch, Rolf A. Herb

**Affiliations:** ^1^Roche Diagnostics International Ltd., Rotkreuz, Switzerland; ^2^Roche Diagnostics GmbH, Penzberg, Germany

**Keywords:** SARS-CoV-2, viral aerosol, HBsAg, laboratory operator, laboratory workflow, surrogate marker

## Abstract

**Objectives:**

To assess aerosol formation during processing of model samples in a simulated real-world laboratory setting, then apply these findings to severe acute respiratory syndrome coronavirus 2 (SARS-CoV-2) to assess the risk of infection to laboratory operators.

**Design:**

This study assessed aerosol formation when using cobas e analyzers only and in an end-to-end laboratory workflow. Recombinant hepatitis B surface antigen (HBsAg) was used as a surrogate marker for infectious SARS-CoV-2 viral particles. Using the HBsAg model, air sampling was performed at different positions around the cobas e analyzers and in four scenarios reflecting critical handling and/or transport locations in an end-to-end laboratory workflow. Aerosol formation of HBsAg was quantified using the Elecsys^®^ HBsAg II quant II immunoassay. The model was then applied to SARS-CoV-2.

**Results:**

Following application to SARS-CoV-2, mean HBsAg uptake/hour was 1.9 viral particles across the cobas e analyzers and 0.87 viral particles across all tested scenarios in an end-to-end laboratory workflow, corresponding to a maximum inhalation rate of <16 viral particles during an 8-hour shift.

**Conclusion:**

Low production of marker-containing aerosol when using cobas e analyzers and in an end-to-end laboratory workflow is consistent with a remote risk of laboratory-acquired SARS-CoV-2 infection for laboratory operators.

## Introduction

Coronavirus disease 2019 is caused by severe acute respiratory syndrome coronavirus 2 (SARS-CoV-2) (1). Widespread testing for SARS-CoV-2 helps prevent the spread of disease by identifying people in the early stages of infection, facilitating disease surveillance, and ensures appropriate, timely treatment for patients if required (2, 3). This highlights the importance of laboratory operators carrying out tests for SARS-CoV-2 as key workers in the context of the current pandemic; however, operators could be at a heightened risk of SARS-CoV-2 infection due to exposure to potentially infectious specimens (4).

Aerosols and droplets are the main forms of transmission of SARS-CoV-2 (5); therefore, practices should be performed in a way to minimize aerosol and droplet formation to reduce the risk of infection to laboratory operators (6–8). There is no clear understanding of infection risk in the laboratory setting (9) and aerosol formation during processing of potentially infectious samples within a SARS-CoV-2 context has yet to be investigated.

The Elecsys^®^ SARS-CoV-2 Antigen test (Roche Diagnostics International Ltd, Switzerland) is an electrochemiluminescence immunoassay used for *in vitro* qualitative detection of the SARS-CoV-2 nucleocapsid protein in nasopharyngeal and oropharyngeal swab samples. The procedure involves the processing of potentially infectious SARS-CoV-2 samples on cobas e analyzers (Roche Diagnostics International Ltd), often as part of an automated end-to-end laboratory workflow. The end-to-end laboratory workflow involves key processes of pre-analytical sample preparation, sample transport to analyzers and automated output or archive solutions, where laboratory operators could be at risk of SARS-CoV-2 infection (9).

One challenge of investigating aerosol formation in a SARS-CoV-2 context is identifying a surrogate marker for SARS-CoV-2-positive samples. Recombinant hepatitis B surface antigen (HBsAg) is a suitable surrogate as previous studies have shown that it self-assembles to form virus-like particles that are assumed to distribute *via* aerosols, which allows comparison with typical airborne infectious agents (10, 11). Recombinant HBsAg is also comparable, in order of magnitude, to the size of the SARS-CoV-2 viral particle (10, 12). Furthermore, highly positive non-infectious HBsAg samples are stable over time and HBsAg can be detected with very high sensitivity over a wide dynamic range using the Elecsys HBsAg II quant II immunoassay (Roche Diagnostics International Ltd).

The aim of this study was to assess aerosol formation when processing potentially infectious samples using an HBsAg model on a panel of cobas e analyzers and in an automated end-to-end laboratory workflow, similar to the conditions in which the Elecsys SARS-CoV-2 Antigen test would be run. The model was then used to assess the risk to laboratory operators of SARS-CoV-2 infection due to the formation of contaminated aerosols.

## Materials and methods

Two sub-studies assessed aerosol formation when handling potentially infectious samples using a panel of cobas e analyzers (conducted at Roche Diagnostics GmbH, Penzberg, Germany) and when using these analyzers in an end-to-end laboratory workflow (conducted at Roche Diagnostics International Ltd).

### The HBsAg model system

Recombinant HBsAg provided as quality control material for the Elecsys HBsAg II quant II immunoassay kit (both Roche Diagnostics International Ltd) was used as a marker for aerosol formation. Two different batches of HBsAg in phosphate buffered saline (prepared in-house: 137 mM NaCl; 2.7 mM KCl; 10 mM Na_2_HPO_4_; 1.8 mM KH_2_PO_4_) were prepared for each sub-study. The highly positive HBsAg samples had a concentration of 1.2–2.5 x10^6^ IU/mL (determined by measurements of dilution series across the panel of cobas e analyzers) in the cobas e analyzer panel sub-study and a concentration of ~1x10^6^ IU/mL (determined by measurement of a dilution series on a cobas e 801 analytical unit) in the end-to-end laboratory workflow sub-study. In the cobas e analyzer panel sub-study, 10% of samples were HBsAg-highly positive and the remainder were HBsAg negative. This simulated a scenario reflective of the situation in German clinical laboratories processing samples for SARS-CoV-2 testing at the time the study was conducted (December 2020). In the end-to-end laboratory workflow sub-study, only HBsAg-highly positive samples were used. For extraction purposes, HBsAg-negative and highly positive samples contained an equivalent volume of HBsAg-specific diluent ([HBSAGQ2 Dil HepB] from the Elecsys HBsAg II quant II immunoassay kit and were used to exclude unknown contamination within the experimental setup and laboratory environment. The negative sample in Elecsys SARS-CoV-2 assay runs was in-house viral transport medium (final concentration: 2% fetal bovine serum, 100 μg/mL gentamycin and 0.5 μg/mL amphotericin B in sterile Hanks balanced salt solution 1X with calcium and magnesium ions; Roche Diagnostics, Germany; not commercially available). To establish a baseline for HBsAg measurements, blank filters were eluted and then quantified with the Elecsys HBsAg II quant II immunoassay.

### Aerosol formation during use of cobas e analyzers

Prior to the main experiments, two air sampling plausibility checks (Supplementary material; Supplementary Figure 1) were carried out in an open, aerosol-allowing environment. This was for the purposes of demonstrating that (i) intense handling of HBsAg-highly positive samples can produce HBsAg aerosol particles and (ii) that the air sampling technique used in all experiments was able to capture HBsAg aerosol particles, including the elution of HBsAg with HBsAg-specific diluent (HBSAGQ2 Dil HepB) from the filters and quantification with the Elecsys HBsAg II quant II immunoassay.

Aerosol formation was investigated on the cobas e 801 analytical unit (cobas^®^ 8000 configuration), cobas e 402 analytical unit, cobas e 601 module, cobas e 411 analyzer and the cobas e 801 in combination with cobas pro integrated solution (quattro-BB-configuration, equipped with four cobas e 801 analytical units) analyzers; these units were selected because they all include testing modules for respiratory pathogens. All of the analyzers used in the study were fully automated with no manual steps; therefore, the difference in reproducibility between runs was negligible. Samples were processed using the pipetting sequences and application of the Elecsys SARS-CoV-2 Antigen test. All cobas e analyzers were operated under routine laboratory conditions at their respective maximum throughput and full operation speed for 4.5–8.3 h. A control run with negative samples only was conducted prior to a single experimental run per analyzer.

Air sampling was conducted using Gilian GilAir Plus Air Sampling Pumps (Sensidyne, USA) equipped with polycarbonate filters (0.8 μm pore size; SKC Inc., Omega Division, USA). The pumps were operated at a sampling air stream of typically 2,000 mL/min (considered by the investigators to be a representative model for human air intake in a closed room) according to manufacturer's instructions for 4.5–8.3 h, in combination with the IOM sampler (SKC Inc.), and were calibrated at the beginning of each day with the Mesalabs Defender 520-M (Mesa Laboratories Lakewood, USA). The air sampling devices were placed around the fan outlets of the cobas e analyzers at shoulder/head height of the laboratory operator and at further distances from the cobas e analyzers through placement around the different laboratory rooms and computer workstations (Supplementary Table 1).

After a fixed time of air sampling (Supplementary Table 1), the content of the filters was eluted with 1 mL HBsAg-specific diluent (HBSAGQ2 Dil HepB). Efficient elution of retained material from filters was experimentally checked prior to commencing the experimental run (Supplementary material; Supplementary Table 2).

The HBsAg concentration of a single sample of eluant per air sampling site was quantified using the Elecsys HBsAg II quant II immunoassay on the cobas e 411 analyzer and was standardized for comparable performance. The effective concentration of HBsAg was determined using the unprocessed signal results from the cobas e 801 analytic unit in conjunction with the corresponding calibration data. In this study, the limit of detection (LoD) and the limit of blank for the Elecsys HBsAg II quant II immunoassay were 0.00929 IU/mL and 0.00517 IU/mL, respectively.

Under routine laboratory conditions, swab testing was performed for all cobas e analyzers included in the panel, except the cobas e 801 analytical unit in combination with cobas pro integrated solution, to assess surface contamination due to aerosol formation (Supplementary Figure 2). Five areas around each analyzer were swabbed with dry separation sheets of the polycarbonate air sampler filters. To replicate a worst-case scenario, swab testing assessed the accumulation of HBsAg following 8 h of full operation without any cleaning. The elution and quantification of HBsAg was performed as described above.

### Aerosol formation in an end-to-end laboratory workflow

**Figure 1:**
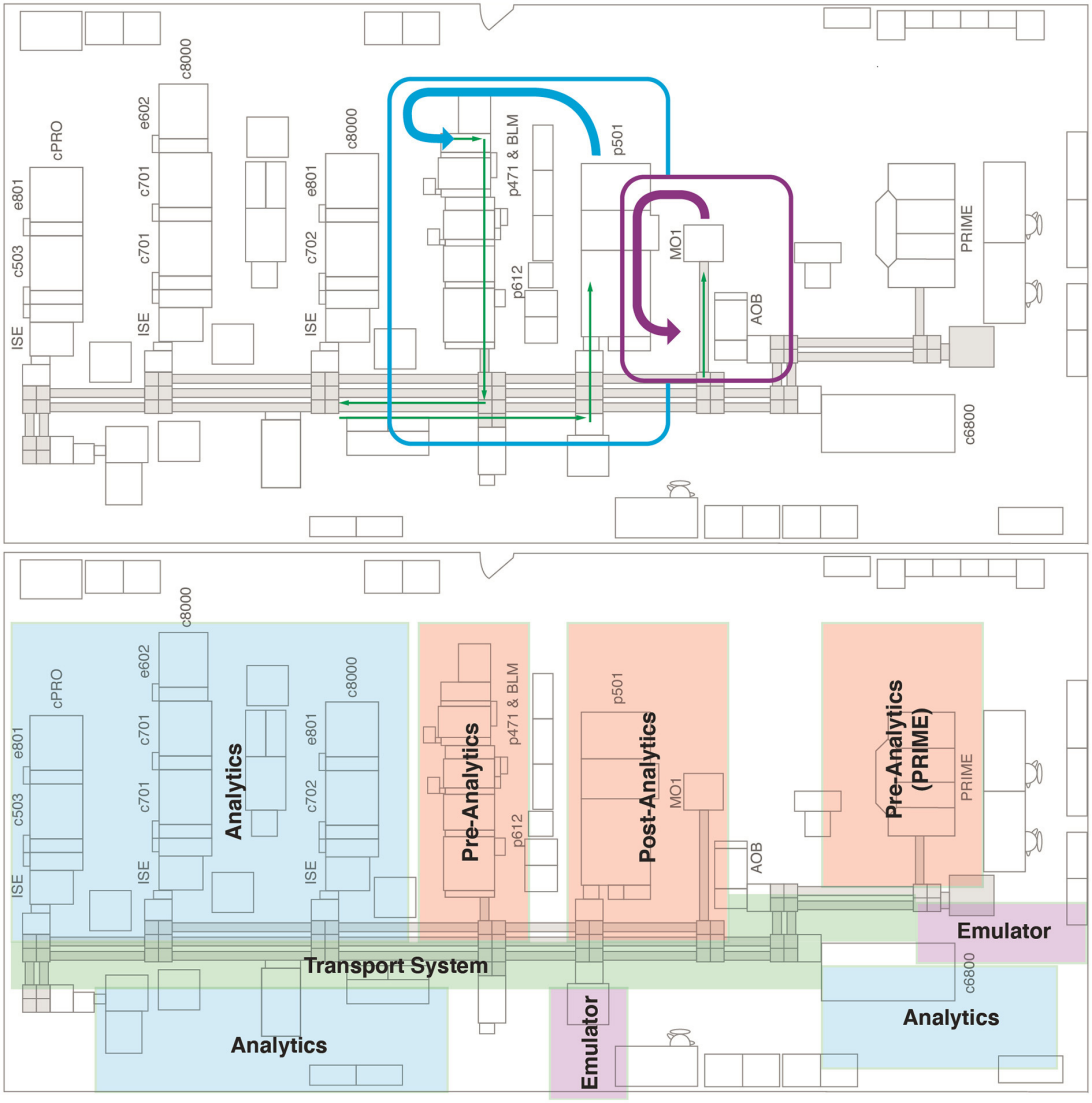
Floor plan of the end-to-end laboratory workflow sub-study. Blue, purple, and green arrows indicate the manual transport of sample tubes. AOB, add-on buffer unit; BLM, bulk loader module; cPRO, cobas pro; c503, cobas 503 clinical chemistry analyzer; c701, cobas 701 clinical chemistry analyzer; c702, cobas 702 clinical chemistry analyzer; c6800, cobas 6800 molecular analyzer; c8000, cobas 8000 modular analyzer; e602, cobas 602 immune analyzer; e801, cobas e 801 analytical unit; ISE, ion selective electrode module; MO1, manual output station 1; PRIME, cobas PRIME, pre-analytical system; p471, cobas p 471 centrifuge unit; p501, cobas p 501 post-analytical system; p612, cobas p 612 pre-analytical unit.

**Figure 2:**
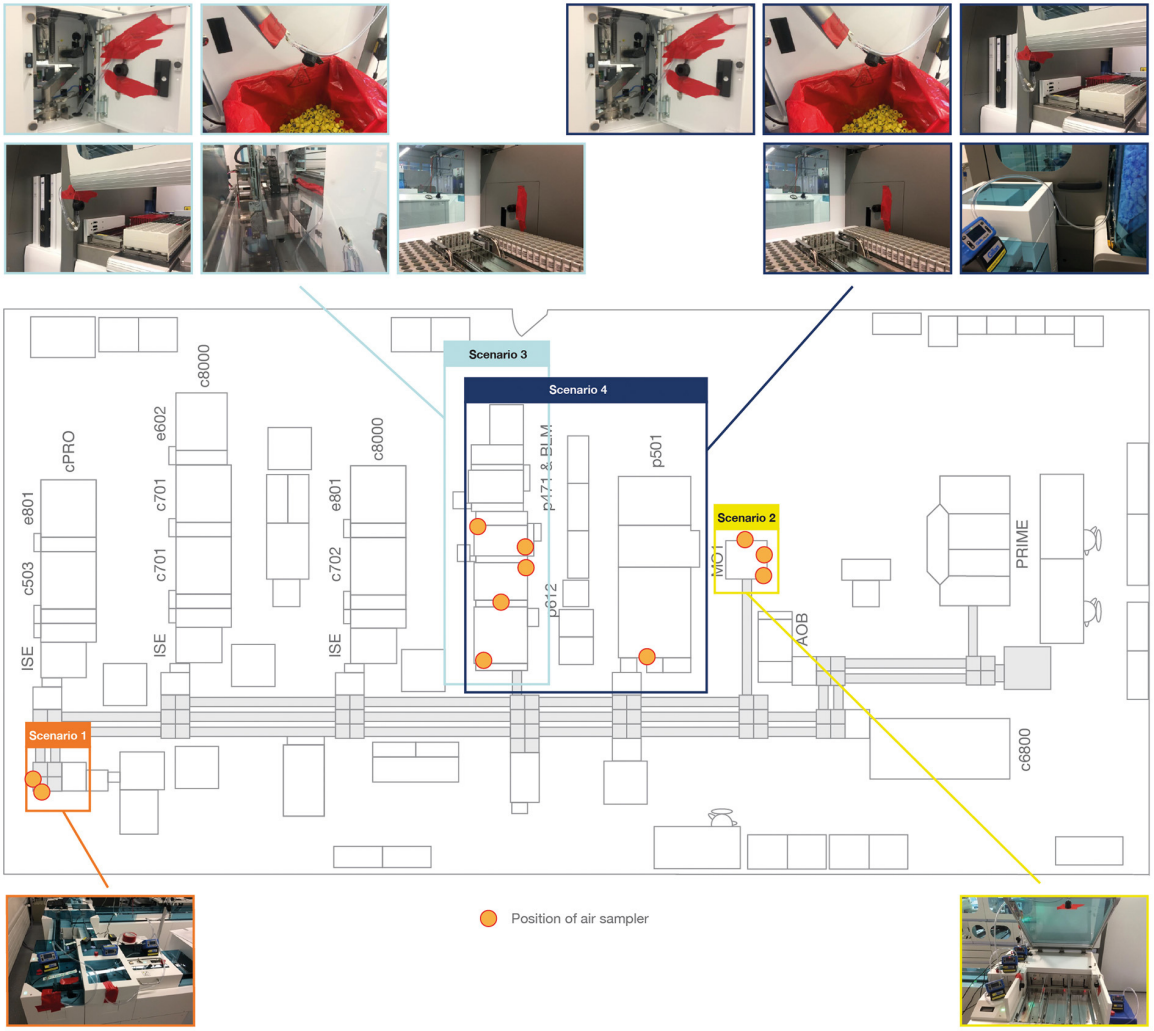
Placement of air sampling units in the end-to-end laboratory workflow. AOB, add-on buffer unit; BLM, bulk loader module; cPRO, cobas pro; c503, cobas 503 clinical chemistry analyzer; c701, cobas 701 clinical chemistry analyzer; c702, cobas 702 clinical chemistry analyzer; c6800, cobas 6800 molecular analyzer; c8000, cobas 8000 modular analyzer; e602, cobas 602 immune analyzer; e801, cobas e 801 analytical unit; ISE, ion selective electrode module; MO1, manual output station 1; PRIME, cobas PRIME, pre-analytical system; p471, cobas p 471 centrifuge unit; p501, cobas p 501 post-analytical system; p612, cobas p 612 pre-analytical unit.

Aerosol formation in an end-to-end laboratory workflow was assessed across four different test scenarios (Supplementary Table 3) that reflect critical processes and locations within this workflow (Figures 1, 2). In brief, the instruments assessed were the cobas connection modules (scenario 1; Roche Diagnostics International Ltd), manual output station 1 (scenario 2 and 4b; Hitachi, Japan), the cobas p 612 pre-analytical unit (scenario 3 and 4; Roche Diagnostics International Ltd), and the cobas p 501 post-analytical unit (scenario 4a; Roche Diagnostics International Ltd).

The test scenarios were chosen to allow assessment of aerosol formation during pre-analytical sample preparation, sample transport to the analyzers and automated output or archive solutions. Within the test scenarios, different sample tubes (Supplementary Table 2) were used to represent the different closure types and the most common sample tube dimensions. In scenarios 1 and 2, samples tubes were also selected for their relatively small filling volume, which allows a high filling level with a limited amount of HBsAg solution. A TeraTerm (Hitachi, Japan) was used in scenarios 1 and 2 to enable processing of 5-position racks directly on a linear conveyor without the use of the cobas p 612 pre-analytical unit as an entry point for the sample tube to the transport system. Similar to the cobas e analyzer panel sub-study, samples were processed using the pipetting sequences and application of the Elecsys SARS-CoV-2 Antigen test.

The air sampling pump and operating procedure described above for the cobas e analyzer panel sub-study was also applied to this sub-study. Air sampling devices were positioned to measure aerosol formation at handling and transport locations (Figure 2). Following air sampling, the elution and quantification of HBsAg was performed as described above for the cobas e analyzer panel sub-study but was conducted on the cobas e 801 analytical unit.

### Application to a SARS-CoV-2 context

The quantified HBsAg values were applied to a SARS-CoV-2 context using the following assumptions: the typical air intake of a human is 600 L/h; the HBsAg-highly positive sample used in the experimental model represents a sample from a highly infectious SARS-CoV-2 individual with a viral load of 5 x 10^8^ viral particles/mL (13); the effective dose needed to cause infection by SARS-CoV-2 is between 100–1,000 viral particles/mL (13).

For air sampling in both studies, the HBsAg uptake for an individual was calculated by dividing a typical human air intake of 600 L/hour by the average volume of air drawn through all air sampling filters per analyzer and then multiplied by the HBsAg reduction factor (calculated by dividing the concentration of aerosol HBsAg particles measured during air sampling by the HBsAg-highly positive sample concentration). The HBsAg uptake for an individual, in terms of viral particles, was then calculated by multiplying the HBsAg uptake for an individual by the viral load (assumed to be 5 x 10^8^ viral particles/mL). In the end-to-end laboratory workflow sub-study, as only samples containing HBsAg were used, the HBsAg uptake by an individual, in terms of viral particles, was divided by 10 as it was assumed that 10% of the SARS-CoV-2 samples in a laboratory would be positive; therefore, mirroring the cobas e analyzer panel sub-study. An overview of the calculations used for air sampling in the study is provided in Table 1. For swab testing, the estimated maximum viral particles were calculated by multiplying the HBsAg reduction factor (calculated in the same way as the air sampling data) by 5 x 10^8^ (representing a viral load of 5 x 10^8^ viral particles/mL; Table 2).

**Table 1:** Overview of the calculations and results for air sampling^*^.

**Overview of experimental results**	**cobas e analyzer panel sub-study**					**End-to-end laboratory workflow sub-study**			
	**cobas e 801 (cobas 8000 configuration)**	**cobas e 402**	**cobas e 601**	**cobas e 411**	**cobas e 801 with cobas pro integrated solution**	**Scenario 1**	**Scenario 2**	**Scenario 3**	**Scenario 4**
HBsAg concentration (IU/mL)^†^	0.01	0.00167	0.003	0.004	0.004	0.04975	0.00244	0.00585	0.02074
Corrected maximum HBsAg concentration^‡^ (IU/mL)	0.01	0.01	0.01	0.01	0.01	N/A	N/A	N/A	N/A
A) HBsAg concentration used in calculation (IU/mL)	0.01	0.01	0.01	0.01	0.01	0.04975	0.00244	0.00585	0.02074
B) Positive HBsAg sample concentration (IU/mL)	~2 x 10^6^	~2.5 x 10^6^	~1.2 x 10^6^	~2 x 10^6^	~1.5 x 10^6^	1 x 10^6^	1 x 10^6^	1 x 10^6^	1 x 10^6^
C) HBsAg reduction factor (*C* = A/B)	5 x 10^−9^	4 x 10^−9^	8.3 x 10^−9^	5 x 10^−9^	6.7 x 10^−9^	49.75 x 10^−9^	2.44 x 10^−9^	5.85 x 10^−9^	20.74 x 10^−9^
D) Average volume of air through each filter (L)	900	998	1,900	700	627	1,800	360	240	360
E) typical human air intake	10 L/minute or 600 L/h								
F) HBsAg-uptake of a human per hour (h^−1^) (*F* = C x E/D)	3.3 x 10^−9^	2.4 x 10^−9^	2.6 x 10^−9^	4.3 x 10^−9^	6.4 x 10^−9^	16.58 x 10^−9^	4.07 x 10^−9^	14.62 x 10^−9^	34.57 x 10^−9^
Mean HBsAg-uptake of a human per hour (h^−1^)	3.8 x 10^−9^					17.46 x 10^−9^			
G) HBsAg-uptake of a human per hour recalculated into viral particles (*G* = F x 5 × 10^8^)	1.7	1.2	1.3	2.14	3.2	8.29	2.03	7.31	17.28
HBsAg-uptake of a human per hour recalculated into viral particles in a simulated real-world laboratory setting^¶^	1.7	1.2	1.3	2.14	3.2	0.83	0.20	0.73	1.73
Mean	1.9 viral particles per hour					0.87 viral particles per hour			
	This corresponds to a maximum personal inhalation					This corresponds to a maximum personal inhalation			
	rate of < 16 viral particles in 8 h					rate of < 16 viral particles in 8 h			

HBsAg, hepatitis B surface antigen; IU, international unit; N/A, not applicable.

^*^The differences in the decimal places shown in this table is due to the different readout capabilities of the analyzers.

^†^In the cobas e analyzer panel sub-study, the maximum HBsAg concentration was recorded, whereas the mean HBsAg concentration was recorded in the end-to-end laboratory workflow sub-study.

^‡^A maximum value of 0.01 was chosen as a safety margin as it was equal to or above all measured HBsAg concentrations.

^¶^To apply the results to a worst-case simulated real-world laboratory setting (10% of SARS-CoV-2 samples submitted to a laboratory are positive), 10% of samples contained HBsAg marker in the cobas e analyzer panel sub-study; whereas, in the end-to-end laboratory workflow sub-study, the assumption that 10% of the SARS-CoV-2 samples in a laboratory are positive was applied when calculating the uptake of viral particles.

**Table 2:** Overview of the calculations and results for swab testing^*,†^.

**Overview of experimental results**	**cobas e analyzer panel sub-study**							
	**cobas e 801**		**cobas e 402**		**cobas e 601**		**cobas e 411**	
	**(cobas 8000 configuration)** ^**‡**^							
	**Before HBsAg assay run**	**After HBsAg assay run**	**Before HBsAg assay run**	**After HBsAg assay run**	**Before HBsAg assay run**	**After HBsAg assay run**	**Before HBsAg assay run**	**After HBsAg assay run**
A) HBsAg concentration (IU/mL)^¶^	-	0.102	0.0024	0.0016	0.0024	0.022	0.004	0.004
B) Positive HBsAg sample concentration (IU/mL)	~2 x 10^6^	~2 x 10^6^	~2.5 x 10^6^	~2.5 x 10^6^	~1.2 x 10^6^	~1.2 x 10^6^	~2 x 10^6^	~2 x 10^6^
C) HBsAg reduction factor (C = A/B)^¶^	-	51.0 x 10^−9^	0.96 x 10^−9^	0.64 x 10^−9^	2.0 x 10^−9^	18.3 x 10^−9^	2.0 x 10^−9^	2.0 x 10^−9^
Estimated maximum viral particles (C x 5 × 10^8^)^¶^	-	25.5	0.48	0.32	1.0	9.2	1.0	1.0

HBsAg, hepatitis B surface antigen; IU, international unit.

^*^Swab testing assessed the accumulation of HBsAg over 1 day of full operation without any cleaning to replicate a worst-case scenario.

^†^The differences in the decimal places shown in this table is due to the different readout capabilities of the analyzers.

^‡^Data were not collected before the HBsAg assay run for the cobas e 801 analytical unit (cobas 8000 configuration).

^¶^ For each cobas e analyzer, the values presented are the mean results from all different swab testing locations.

### Data processing and analysis

Internally validated software (OASEpro HetIA, Roche Diagnostics International Ltd) was used to calculate the concentration (IU/mL) of HBsAg from the signal detected by the cobas e analyzers and data were processed using Microsoft Excel (Microsoft, USA).

## Results

### cobas e analyzer panel sub-study

**Figure 3:**
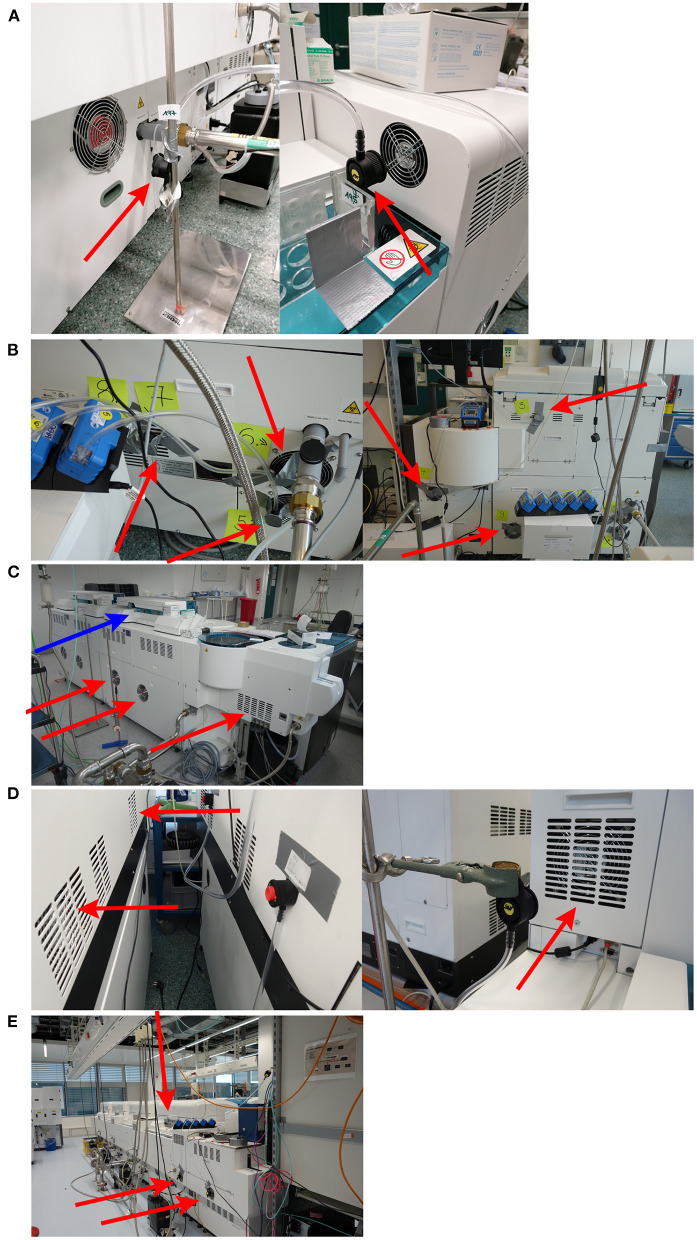
Selection of air sampling positions included in the cobas e analyzer panel sub-study*. **(A)** cobas e 801 analytical unit (cobas 8000 configuration). **(B)** cobas e 402 analytical unit **(C)** cobas e 601 module. **(D)** cobas e 411 analyzer. **(E)** cobas e 801 analytical unit in combination with cobas pro integrated solution. *Positions of air sampling around fan outlets are indicated by red arrows and the sample pipette location is indicated by a blue arrow.

Air sampling points were set up at various positions around the cobas e analyzers, as shown in Figure 3. The maximum concentration of aerosol HBsAg particles was 0.01 IU/mL (recorded from a filter positioned around a fan outlet of the cobas e 801 analytical unit; Table 1). To improve the robustness of the study, 0.01 IU/mL was used as the maximum concentration of HBsAg particles for all cobas e analyzers when calculating the HBsAg reduction factor (Table 1). The cobas e 801 analytical unit in combination with cobas pro integrated solution recorded the highest HBsAg uptake per hour and the highest HBsAg uptake per hour when recalculated into viral particles (6.4 ppb/h and 3.2 particles, respectively; Table 1). The cobas e 402 analytical unit recorded the lowest HBsAg uptake per hour and the lowest HBsAg uptake per hour when recalculated into viral particles (2.4 ppb/h and 1.2 particles, respectively; Table 1). Across all cobas e analyzers, the mean HBsAg uptake per hour when recalculated into viral particles was 1.9 particles, which results in a maximum inhalation rate of <16 viral particles during a typical 8-h shift for a laboratory operator (Table 1).

Swab testing around the cobas e 801 analytical unit, after the HBsAg assay run, recorded the highest mean estimated maximum viral particles (25.5 particles; Table 2). Swab testing around the cobas e 402 analytical unit, after the HBsAg assay run, recorded the lowest mean estimated maximum viral particles (0.32 particles; Table 2). For the cobas e 402 analytical unit, cobas e 601 module and cobas e 411 analyzers, there was minimal difference in the mean estimated maximum viral particles before and after the HBsAg assay run (Table 2).

### End-to-end laboratory workflow sub-study

**Figure 4:**
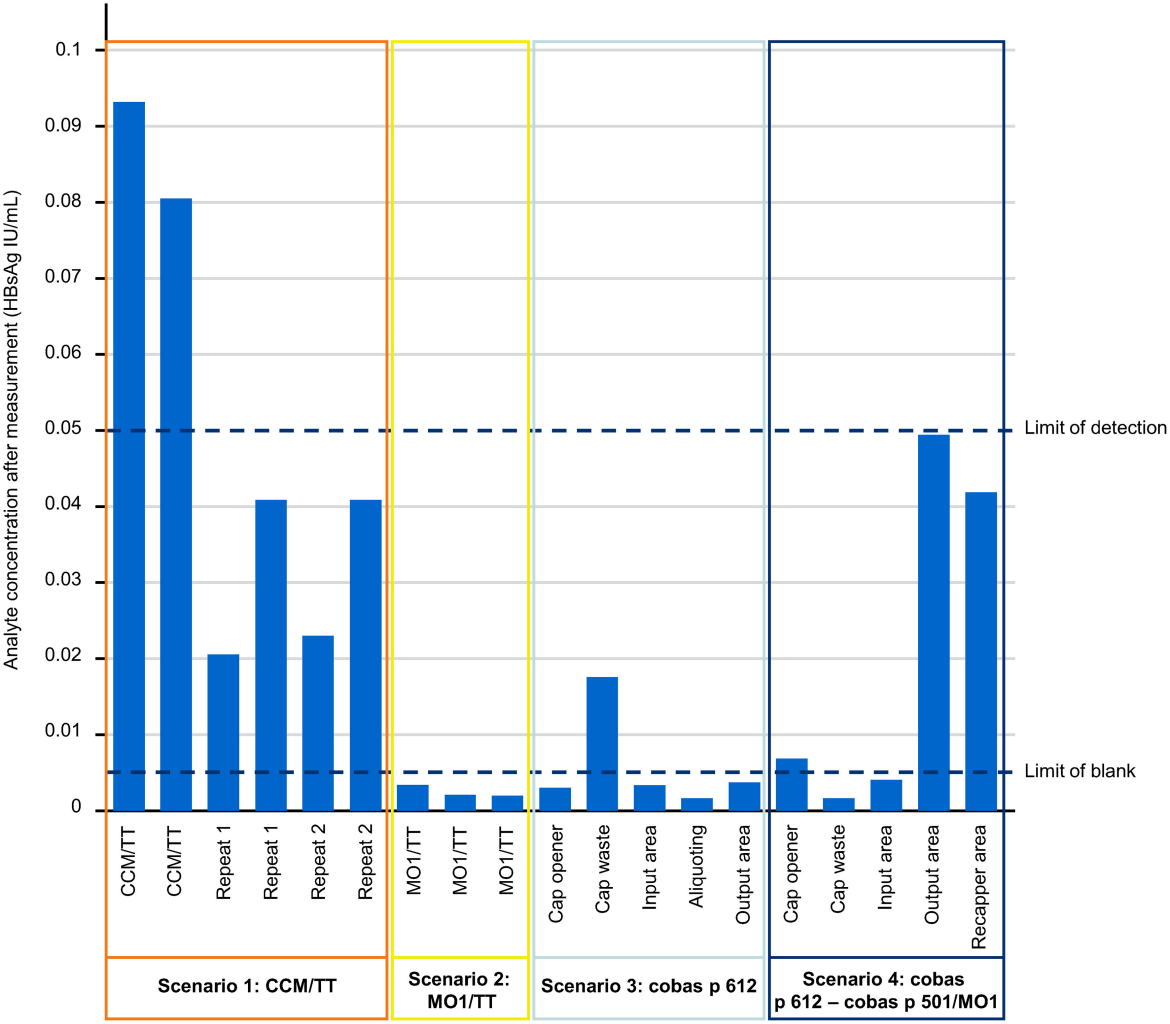
The effective HBsAg analyte concentration from each of the testing scenarios investigated in the end-to-end laboratory workflow sub-study. Quantification of HBsAg was performed on the cobas e 801 analytical unit. CCM, cobas connection module; HBsAg, hepatitis B surface antigen; IU, international unit; MO1, manual output station 1; TT, TeraTerm.

Air sampling points were set up at various positions across the four test scenarios, as shown in Supplementary Figure 3. In scenario 1 only, the eluate from the extracted filters exhibited an effective concentration of HBsAg particles above the LoD (0.05 IU/mL) of the Elecsys HBsAg II quant II immunoassay (Figure 4). For scenario 1, HBsAg levels above the LoD were also evident in the corresponding negative control and this result was not observed in the subsequent repetitions of the experiment (Figure 4).

Scenario 1 produced the highest mean concentration of aerosol HBsAg particles (0.04975 IU/mL) across the tested scenarios (Table 1). Scenarios 4 and 2 produced the highest and lowest HBsAg uptake per hour (34.57 ppb/h and 4.07 ppb/h, respectively; Table 1). With the assumption that 10% of the SARS-CoV-2 samples in a laboratory are positive, scenarios 4 and 2 also produced the highest and lowest HBsAg uptake per hour when recalculated into viral particles (1.73 particles and 0.20 particles, respectively; Table 1), while the corresponding mean across the test scenarios was 0.87 particles (Table 1). As such, there was a mean maximum inhalation rate of <16 viral particles during an 8-h shift.

## Discussion

To our knowledge, this is the first study to assess aerosol formation when processing potentially infectious samples using a panel of cobas e analyzers and in an automated end-to-end laboratory workflow. In this study, data on aerosol formation were applied to a SARS-CoV-2 context and the low number of viral particles detected inferred a remote risk of SARS-CoV-2 infection to laboratory operators when processing potentially infectious samples.

In scenario 1 (the cobas connection module/TeraTerm units) of the end-to-end laboratory workflow sub-study, elevated levels of HBsAg above the LoD were evident. Elevated levels were also evident in the corresponding negative control; however, such levels were not present in the subsequent repetitions of the experiment. This can be considered a contamination-driven effect and was not considered to originate from the samples processed on the cobas connection module/TeraTerm units.

HBsAg can self-assemble to virus-like particles that are assumed to distribute *via* aerosols; therefore, the use of recombinant HBsAg in the model system allows the model to be applied to all airborne pathogens and not just SARS-CoV-2. This addresses an important unmet clinical need as aerosol formation has previously been undervalued in terms of transmission of respiratory viral diseases due to a lack of understanding of how infectious aerosols are produced and transported (14).

In this study, the mean maximum inhalation rate detected was <16 viral particles during an 8-h shift when using cobas e analyzers and in an end-to-end laboratory context. Furthermore, for swab testing, the greatest number of estimated maximum viral particles was limited to 25.5 particles over 8 h of full operation and there was only a minimal difference in mean estimated maximum viral particles before and after the HBsAg assay run. The infectious dose of a virus can vary greatly among respiratory viruses (15). For SARS-CoV-2, the minimum dose of viral particles necessary to cause infection remains an active research question; however, it has been estimated to be between 100 and 1,000 particles (16–19). Therefore, the risk of SARS-CoV-2 infection for laboratory operators when using cobas e analyzers and in an end-to-end laboratory workflow can be considered remote. Any potential cross-contamination of other samples resulting from the aerosol particles is out of the scope of the current study; however, given the low number of viral particles detected cross-contamination would be unlikely. Applying the outcomes of studies of other viruses that have evaluated risk of infection to laboratory operators may not be relevant in a SARS-CoV-2 context due to the highly infectious nature of SARS-CoV-2; generally inhaled viruses require 1,950–3,000 particles to cause infection, compared with between 100 and 1,000 particles for SARS-CoV-2 (16–19).

When applying the findings of the study to a SARS-CoV-2 context, the assumption regarding viral load did not consider the effect of variants of SARS-CoV-2. For example, in the first known community transmission event of the delta variant in mainland China, it was shown that the viral load associated with the delta variant was ~1,000 times higher than with the alpha or beta strain (20). In addition, vaccination status has also been shown to affect viral load kinetics, whereby vaccinated individuals have a faster mean rate of viral load decline relative to unvaccinated individuals (21). Some European countries have already made vaccination against coronavirus disease 2019 mandatory for all healthcare workers (22, 23).

A strength of this study is that a worst-case scenario (10% of samples were highly positive) was tested in several different analyzers using a clinically relevant end-to-end laboratory setup; thus, simulating a real-world testing laboratory for SARS-CoV-2. A further strength of this study is that swab testing of the instrument surfaces was performed, which, in addition to aerosol formation, could represent a source of infection for laboratory workers (24–27). In a similar study, Farnsworth et al. (28) used a fluorescent marker to assess instrument and specimen contamination and found that while there is a low risk of instrument contamination, the handling of infectious specimen containers can contaminate laboratory surfaces. Relative to a fluorescent marker, the use of the HBsAg model in this study was more representative of the mass:volume ratio seen in the aerosol distribution of viral particles. Correct adherence in the use of personal protective equipment can be very effective in preventing infection through contact with contaminated surfaces (28) and inhalation of droplets associated with SARS-CoV-2 infection (29, 30). Van Doremalen et al. (8) found that under experimental conditions, SARS-CoV-2 is viable in aerosols for 3 h; therefore, personal protective equipment should always be worn in a laboratory setting. Healthcare workers caring for patients with coronavirus disease 2019 report adherence rates of 98.6% to personal protective equipment protocols (31); similar adherence rates are important to reduce SARS-CoV-2 infection risk in a laboratory context.

There are stability differences between HBsAg and SARS-CoV-2 and the ability of HBsAg to self-assemble may affect data interpretation; however, using recombinant HBsAg also means that this study can potentially be used to estimate the risk to laboratory workers of airborne infectious agents other than SARS-CoV-2. Future studies could consider using heat- or chemically inactivated SARS-CoV-2 virus instead of HBsAg and should assess molecular modules, to expand upon the findings for analyzers shown here. Future studies could also consider using nucleic acid amplification techniques to obtain a better estimate of the number of viral particles produced.

In conclusion, the low production of potentially infectious aerosols when using cobas e analyzers and in an end-to-end laboratory workflow (which includes processes of pre-analytical sample preparation, sample transport to analyzers and automated output or archive solutions) is consistent with a remote risk of SARS-CoV-2 infection for laboratory operators. These results can be considered reassuring to laboratory operators involved in the processing of potentially infectious samples.

## Data availability statement

The data supporting this manuscript can be requested in writing from the corresponding author.

## Author contributions

GB, AL, and RH contributed to study conception/design, data acquisition, and data analysis/interpretation. ME, OL, and BO-H contributed to study conception/design and data analysis/interpretation. DH and MR contributed to data acquisition and data analysis/interpretation. All authors contributed to writing and/or critical review and approved the final version for submission.

## Funding

The cobas e analyzer panel sub-study was funded by Roche Diagnostics GmbH (Penzberg, Germany). Roche Diagnostics International Ltd., (Rotkreuz, Switzerland) funded the end-to-end laboratory workflow sub-study and third-party medical writing support.

## Conflict of interest

Employment or Leadership: Author GB is employed by Roche Diagnostics International Ltd. Authors ME, DH, OL, AL, BO-H, MR, and RH are employed by Roche Diagnostics GmbH. Consultant or Advisory Role: Author ME participated in the Roche Diagnostics SARS-CoV-2 Biosafety, Antigen Testing and Infectivity Advisory Board Meeting (8th December 2020). Stock Ownership: Authors GB, ME, OL, BO-H, and RH hold non-voting equities in F. Hoffmann-La Roche.

## Publisher's note

All claims expressed in this article are solely those of the authors and do not necessarily represent those of their affiliated organizations, or those of the publisher, the editors and the reviewers. Any product that may be evaluated in this article, or claim that may be made by its manufacturer, is not guaranteed or endorsed by the publisher.

## References

[1] Zhou P Yang XL Wang XG Hu B Zhang L Zhang W. A pneumonia outbreak associated with a new coronavirus of probable bat origin. Nature. (2020) 579:270–3. 10.1038/s41586-020-2012-7 32015507 PMC7095418

[2] Salathé M Althaus CL Neher R Stringhini S Hodcroft E Fellay J. COVID-19 epidemic in Switzerland: on the importance of testing, contact tracing and isolation. Swiss Med Wkly. (2020) 150:w20225. 10.4414/smw.2020.20225 32191813

[3] Lippi G Plebani M. The critical role of laboratory medicine during coronavirus disease 2019 (COVID-19) and other viral outbreaks. Clin Chem Lab Med. (2020) 58:1063–9. 10.1515/cclm-2020-0240 32191623

[4] Pambuccian SE. The COVID-19 pandemic: implications for the cytology laboratory. J Am Soc Cytopathol. (2020) 9:202–11. 10.1016/j.jasc.2020.03.001 32284276 PMC7104051

[5] Wang J Du G. COVID-19 may transmit through aerosol. Ir J Med Sci. (2020) 189:1143–4. 10.1007/s11845-020-02218-2 32212099 PMC7094991

[6] CDC. Interim laboratory biosafety guidelines for handling and processing specimens associated with coronavirus disease 2019 (COVID-19). (2021). Available online at: https://www.cdc.gov/coronavirus/2019-nCoV/lab/lab-biosafety-guidelines.html (accessed October 26, 2021).

[7] WHO. Laboratory biosafety guidance related to coronavirus disease (COVID-19): Interim guidance, 28 January 2021. (2021). Available online at: https://www.who.int/publications/i/item/WHO-WPE-GIH-2021.1 (accessed October 26, 2021).

[8] van Doremalen N Bushmaker T Morris DH Holbrook MG Gamble A Williamson BN. Aerosol and surface stability of SARS-CoV-2 as compared with SARS-CoV-1. N Engl J Med. (2020) 382:1564–7. 10.1056/NEJMc2004973 32182409 PMC7121658

[9] Karthik K Aravindh Babu RP Dhama K Chitra MA Kalaiselvi G Alagesan Senthilkumar TM. Biosafety concerns during the collection, transportation, and processing of COVID-19 samples for diagnosis. Arch Med Res. (2020) 51:623–30. 10.1016/j.arcmed.2020.08.007 32948378 PMC7486853

[10] Yamaguchi M Sugahara K Shiosaki K Mizokami H Takeo K. Fine structure of hepatitis B virus surface antigen produced by recombinant yeast: comparison with HBsAg of human origin. FEMS Microbiol Lett. (1998) 165:363–7. 10.1111/j.1574-6968.1998.tb13171.x 9742710

[11] Ludwig C Wagner R. Virus-like particles-universal molecular toolboxes. Curr Opin Biotechnol. (2007) 18:537–45. 10.1016/j.copbio.2007.10.013 18083549 PMC7126091

[12] Laue M Kauter A Hoffmann T Möller L Michel J Nitsche A. Morphometry of SARS-CoV and SARS-CoV-2 particles in ultrathin plastic sections of infected Vero cell cultures. Sci Rep. (2021) 11:3515. 10.1038/s41598-021-82852-7 33568700 PMC7876034

[13] Lelieveld J Helleis F Borrmann S Cheng Y Drewnick F Haug G. Model calculations of aerosol transmission and infection risk of COVID-19 in indoor environments. Int J Environ Res Public Health. (2020) 17:8114. 10.3390/ijerph17218114 33153155 PMC7662582

[14] Wang CC Prather KA Sznitman J Jimenez JL Lakdawala SS Tufekci Z. Airborne transmission of respiratory viruses. Science. (2021) 373:eabd9149. 10.1126/science.abd9149 34446582 PMC8721651

[15] Di Girolamo P. Assessment of the potential role of atmospheric particulate pollution and airborne transmission in intensifying the first wave pandemic impact of SARS-CoV-2/COVID-19 in Northern Italy. Bull Atmos Sci Technol. (2020) 1:515–50. 10.1007/s42865-020-00024-3 PMC7750914 38624634

[16] Evans MJ. Avoiding COVID-19: aerosol guidelines. medRxiv. (2020). Available online at: https://www.medrxiv.org/content/medrxiv/early/2020/06/05/2020.05.21.20108894.full.pdf

[17] Karimzadeh S Bhopal R Nguyen Tien H. Review of infective dose, routes of transmission and outcome of COVID-19 caused by the SARS-CoV-2: comparison with other respiratory viruses. Epidemiol Infect. (2021) 149:e96. 10.1017/S0950268821001084 33849679 PMC8082124

[18] Barr GD. A model showing the relative risk of viral aerosol infection from breathing and the benefit of wearing masks in different settings with implications for Covid-19. medRxiv. (2020). Available online at: https://www.medrxiv.org/content/medrxiv/early/2020/08/18/2020.04.28.20082990.full.pdf

[19] Basu S. Close-range exposure to a COVID-19 carrier: transmission trends in the respiratory tract and estimation of infectious dose. medRxiv. (2020). Available online at: https://www.medrxiv.org/content/medrxiv/early/2020/07/29/2020.07.27.20162362.full.pdf

[20] Li B Deng A Li K Hu Y Li Z Xiong Q. Viral infection and transmission in a large, well-traced outbreak caused by the SARS-CoV-2 Delta variant. medRxiv. (2021). Available online at: https://www.medrxiv.org/content/medrxiv/early/2021/07/23/2021.07.07.21260122.full.pdf 10.1038/s41467-022-28089-y PMC8786931 35075154

[21] Singanayagam A Hakki S Dunning J Madon KJ Crone MA Koycheva A. Community transmission and viral load kinetics of the SARS-CoV-2 delta (B. 16172) variant in vaccinated and unvaccinated individuals in the UK: a prospective, longitudinal, cohort study. Lancet Infect Dis. (2022) 22:183–95. 10.1016/S1473-3099(21)00648-4 34756186 PMC8554486

[22] Paterlini M. Covid-19: Italy makes vaccination mandatory for healthcare workers. BMJ. (2021) 373:n905. 10.1136/bmj.n905 33824155

[23] Wise J. Covid-19: France and Greece make vaccination mandatory for healthcare workers. BMJ. (2021) 374:n1797. 10.1136/bmj.n1797 34261643

[24] Boone SA Gerba CP. Significance of fomites in the spread of respiratory and enteric viral disease. Appl Environ Microbiol. (2007) 73:1687–96. 10.1128/AEM.02051-06 17220247 PMC1828811

[25] Lopman B Gastañaduy P Park GW Hall AJ Parashar UD Vinjé J. Environmental transmission of norovirus gastroenteritis. Curr Opin Virol. (2012) 2:96–102. 10.1016/j.coviro.2011.11.005 22440972

[26] Repp KK Keene WE. A point-source norovirus outbreak caused by exposure to fomites. J Infect Dis. (2012) 205:1639–41. 10.1093/infdis/jis250 22573873 PMC3415849

[27] Thornley CN Emslie NA Sprott TW Greening GE Rapana JP. Recurring norovirus transmission on an airplane. Clin Infect Dis. (2011) 53:515–20. 10.1093/cid/cir465 21836128

[28] Farnsworth CW Wallace MA Liu A Gronowski AM Burnham C-AD Yarbrough ML. Evaluation of the risk of laboratory microbial contamination during routine testing in automated clinical chemistry and microbiology laboratories. Clin Chem. (2020) 66:1190–9. 10.1093/clinchem/hvaa128 32870987

[29] Dos Santos WM. Use of personal protective equipment reduces the risk of contamination by highly infectious diseases such as COVID-19. Evid Based Nurs. (2021) 24:41. 10.1136/ebnurs-2020-103304 32788168

[30] Verbeek JH Rajamaki B Ijaz S Sauni R Toomey E Blackwood B. Personal protective equipment for preventing highly infectious diseases due to exposure to contaminated body fluids in healthcare staff. Emergencias. (2021) 33:59–61. 10.1002/14651858.CD011621.pub4 33496400

[31] Zhao Y Liang W Luo Y Chen Y Liang P Zhong R. Personal protective equipment protecting healthcare workers in the Chinese epicentre of COVID-19. Clin Microbiol Infect. (2020) 26:1716–8. 10.1016/j.cmi.2020.07.029 32712241 PMC7377698

